# Supplementary material for: Are carbon emissions trading and green financial instruments synergistic? -Comprehensive quantitative research based on content analysis
Source: PLoS One. 2024 Mar 7;19(3):e0298601. doi: 10.1371/journal.pone.0298601 (PMC10919601; doi:10.1371/journal.pone.0298601)
Supplement: S1 Data — (ZIP) [file pone.0298601.s001.zip › Data/地级市Z_F工作报告.zip!/░▓╗╒/│n╓▌╩╨/└┤╫╘╣1⁄2╓┌║┼í╛┬φ┐╦╩2╛▌═°í┐_2013.docx]

各位代表：

　　现在，我代表市人民政府，向市第五届人民代表大会第一次会议报告工作，请予审议，并请市政协委员和其他列���人员提出意见。

　　一、本届政府任期以来工作回顾

　　2008年以来，在省委、省政府和市委的正确领导下，在市人大、市政协的监督支持下，市政府与全市人民一道，以科学发展观为指导，解放思想，苦干实干，圆满完成了市四届人大历次会议确定的任务，推动滁州进入加速赶超、崛起跨越的新时期。

　　五年来，我们全面拉升标杆，奋力冲刺，发展速度日渐加快，整体实力迈上新台阶。

　　全市上下抢抓产业转移和示范区建设等重大机遇，同步推进工业化、城镇化和农业现代化，主要经济指标实现翻番。2012年，全市完成生产总值970.7亿元，比2007年翻一番，年均增长13.4%；财政收入153.2亿元，是2007年的3.4倍，年均增长27.5%；规模以上工业增加值401.3亿元，是2007年的2.9倍，年均增长22.9%；固定资产投资882.6亿元，是2007年的4.95倍，年均增长37.7%；社会消费品零售总额296.8亿元，是2007年的2.3倍，年均增长18.2%；外贸进出口总额15.3亿美元，是2007年的3.2倍，年均增长26.3%；城镇居民人均可支配收入20426元，年均增长13.4%；农民人均纯收入8091元，年均增长16.2%。2010年和2011年，我市在全省年度目标综合考核中分别排名第4位和第3位，受到省政府的通报嘉奖。

　　五年来，我们着力壮大实体经济，突出工业主导，三次产业加速发展，产业结构明显优化。

　　紧紧抓住招商引资增投入、兴建园区筑平台、争取要素强保障三项关键举措，强力推动实体经济发展。五年招商引资3700亿元，是前五年的8倍，美国嘉吉、霍尼韦尔、沃尔玛、台玻、雀巢银鹭、盼盼食品、碧桂园、香江物流、远东集团、华塑盐化等国内外知名企业相继落户。省级以上开发园区从7个增加到15个，规划面积从139平方公里增加到600平方公里，建成区面积从95平方公里增加到180平方公里，实现税收从10亿元增加到38亿元，特别是引进了中新集团共建苏滁现代产业园，市开发区成功创建国家级开发区和省高新区。抢抓国家城乡建设用地增减挂钩试点机遇，开展建设用地置换14万亩，居全省第一；组建政府投融资平台，统筹运作国有资产，解决基础设施建设筹融资问题，为大建设大发展提供了重要保障。

　　工业上，五年完成投资1585亿元，其中技改投资603亿元。市开发区的长电科技、康佳白电和液晶模组、天大钢管二期、定远华塑盐化一期、天长强强实业、明光榄菊日化、来安中联能源、全椒叉车发动机及铸造、凤阳确成硅化工、南谯恒昌机械、琅琊金春无纺布等一批重大项目建成投产。推进大企业上台阶、中企业上水平、小企业上规模，规模以上工业企业发展到1008家，其中亿元以上338家，五年分别增长2.9倍和3.4倍。狠抓科技创新和产学研结合，省级以上高新技术企业由8家发展到86家，高新技术产值占工业总产值的比重由38.3%提高到43.7%，获得全国科技进步市称号。拥有中国驰名商标4件、地理标志证明商标3件、省名牌产品78个。服务业上，一手抓传统服务业提升，一手抓物流、旅游、金融、房地产等现代服务业培育，累计建成连锁农家店3678个，新增限上商贸企业453家、3A级以上景区7家、四星级以上酒店9家，引进股份制银行5家、风投基金2家，开办小额贷款公司22家、融资担保公司12家、村镇银行6家，5个信用联社成功改组为农商行，德力、金禾成功上市。本外币存款余额超千亿元，贷款余额超700亿元，五年分别增长2.3倍和2.5倍。五年房地产投资610亿元、开发面积2500万平方米。农业上，五年全社会水利投资146亿元，连续四年争取上级水利投资全省第一，完成4条中小河流治理和36座中型、653座小型水库除险加固。植树造林43.2万亩。粮食生产实现“七连增”。规模养殖比重超过60%。农机总动力达638万千瓦。土地流转面积141万亩，农民专业合作社发展到2666家，五年分别增长4倍和6倍。市级以上农业产业化龙头企业发展到121家，其中省级以上43家。农产品加工产值增加到368亿元，五年增长2.8倍。2007-2012年，全市三次产业结构从24.6∶41.5∶33.9调整到19.9∶52.3∶27.8，二产比重过半。

　　五年来，我们建设大滁城，力推城镇化，中心城区焕然一新，城乡面貌发生历史性变化。

　　大滁城“131”组团建设全力推进。主城区五年完成政府性投资454亿元，是前五年的7.8倍。城市框架全面拉开。主城东扩南移西拓北上，规划面积扩大到150平方公里，新建续建道路211条376公里，路网覆盖从40平方公里扩大到90平方公里。新区加快拓展。城南新区展露英姿，市经济技术开发区数倍扩容，科教园区、高铁站区、琅琊山旅游区、琅琊新区、南谯新城、苏滁现代产业园、市集中示范园建设全面展开。老城明显变样。完成征迁面积548万平方米，建设安置房781.5万平方米，改造小街巷118条。功能有力提升。市政务中心、滁州大剧院、规划馆、美术馆、文化馆、体育馆、“一校两团”、滁州中学新校区、市民广场、垃圾填埋场、污水处理一厂二厂、自来水三厂四厂等先后建成投入使用，城市供水、排水、供电、通信、广电、供热、供气及管理能力显著提升。打通断头路11条，建成了明光路、清流路两座高架桥，城市出行更加通畅。城市变得更高、更美、更靓。已建成和在建10层以上建筑1100多幢，是2007年的12倍。“六城联创”深入开展，完成9条道路“白改黑”，实施12处亮化工程，建成龙蟠河、丰乐亭、菱溪湖、清流河、南湖二期和三期等9个公园，绿化覆盖率上升到38.8%，成功创建省园林城市。组团发展取得突破。全椒、来安两个县城和汊河新城积极呼应主城区，五年完成投资191亿元。大滁城建设得到了省委、省政府及全省上下的一致好评，成为对内凝聚共识的重要旗帜、对外展示形象的重要窗口。天长、明光、定远、凤阳4个县城比学赶超，老城改造、新区拓展齐头并进，五年完成建设投资333亿元，面积从63平方公里扩大到106平方公里。加强集镇建设，一批重点镇、特色镇镇容镇貌有了崭新变化。全市城镇化率从37.7%提高到45.4%，五年提高近8个百分点。大力推进新农村建设，新建农村居民点799个，入住42万人。美好乡村建设按照“1313”布局全面启动。城乡基础设施建设得到加强。五年完成交通投资213亿元、电力投资38.5亿元、通信设施投资30亿元；开工建设3条高速公路，完成8条国省道改造；建设“村村通”公路6124公里，居全省第三；凤阳港及滁州、定远两个高铁站投入使用。县市污水、垃圾处理设施全部建成运行，节能减排工作连年受到省政府表彰。　　　　　　　　　　　　　　　（下转第二版）

　　五年来，我们对内改革攻坚，对外扩大开放，体制机制不断创新，发展活力进一步增强。

　　基本完成国有集体企业改革。稳步推进事业单位改革，全面施行事业单位绩效工资制度。深化农村综合改革。先后完成集体林权制度、文化体制和基层医疗卫生体制等改革。全面实施工业用地招拍挂制度。完善政府预算和非税收入管理体制。大力推进全民创业，五年新增民营企业1.4万户、个体工商户7.5万户。积极转变政府职能，完成新一轮政府机构改革，深化人事制度改革。建成市县乡三级政务服务中心，建立了基建联合审批、企业登记注册“一表通”、“3+1”联网审批等平台，实施五轮行政审批流程再造，取消和调整行政审批项目121个，创造了影响全省的“滁州效率”，行政性收费在周边地区动态最低。招标采购平台建设和管理走在全省前列，五年招标采购总额超过千亿元，其中市本级612亿元。大力发展外向型经济，五年实际利用外资12.7亿美元，外贸进出口总额稳居全省第五名。滁州海关和检验检疫局如期开关开检。

　　五年来，我们强化公共服务，着力改善民生，社会事业同步发展，人民生活水平大幅提高。

　　优先发展教育。完善教育经费保障机制，五年财政教育支出近145亿元，年均增长15%。全面完成校舍安全工程，加固和重建校舍205万平方米。实施学前教育三年行动计划，巩固提高义务教育和高中教育，学前教育毛入园率和高中毛入学率分别达84%和88%，均高于全省平均水平。在全省首创校车运营模式。支持安徽科技学院和滁州学院建设发展。推进职教大市建设，设立市科教园区，建成职业技术学院新校区，组建城市职业学院和技师学院，大中专学校在校生从2万增加到7.5万。市县两级重点医疗机构加快建设，全面完成基层卫生院和社区卫生服务机构标准化建设，县级公立医院改革全面启动。加强公共文化服务体系建设，五年建设市县两级图书馆、文化馆、博物馆14个，乡镇综合文化站101个，农家书屋1322个，城乡体育健身工程687个，广播电视“村村通”工程2258个。全民健身工作连续四年获得全国先进。实施205项非物质文化遗产保护。连续四届承办中国农歌会均取得圆满成功，并荣获了文化部创新奖特等奖。实现全国双拥模范城四连冠。完成《滁州市志》编纂。完成第六次人口普查。全市人口计生工作取得突破，迈入全省第一方阵。千方百计促就业，创办40个城镇职工、农民工和大学生创业园（街），五年开展职业技能培训20万人次，新增城镇就业28万人。努力推动社会保险扩面提标，职工医疗、养老、工伤、失业、生育保险参保人次达146万，新农合参合率达99.2%，城镇居民基本医疗保险、城乡居民养老保险和最低生活保障实现全覆盖，五年发放各类社保和优抚资金86.3亿元。解决困难群众生产生活问题，改造农村危房2.8万户，开工建设各类保障房9.1万套，安全饮水工程惠及149万农村人口，农村“五保户”集中供养率达50%。民生工程五年投入150亿元，惠及城乡400多万人。积极发展妇女儿童、老年人、残疾人等事业，支持法院和检察院、工会、共青团、科协、工商联、文联、关工委等工作，档案、编制、气象、人防、保密、对台、地震、红十字会等工作全面加强。

　　五年来，我们致力社会建设，创新社会管理，安定团结大局得到有效巩固，滁州社会更加和谐。

　　开展“五级书记带头大走访”和“三民”工程大走访，加强社会管理体系建设，推行社区网格化管理，组建社区“一委一居一办两站”，全面建成市县两级群众工作部和群众接待、维权、法律服务三中心，完善了服务群众的新平台。积极开展平安滁州建设，排查整改安全生产隐患，强化食品药品安全监管和市场物价、产品质量监测，加强社会管理综合治理，加大刑事犯罪打击和扫黄打非力度，推进法律援助和人民调解工作，建立劳动关系“三方四家”协调机制，落实民族宗教和外事侨务政策，建立网络舆情处置机制，完善突发事件应急管理机制，有效维护了社会稳定。

　　五年来，我们坚持依法行政，大力提升效能，自身建设不断加强，服务水平不断提高。

　　自觉接受人大及其常委会的法律监督和工作监督，积极支持政协履行政治协商、民主监督、参政议政职能，五年办理市人大议案、代表建议和政协委员提案1857件，办理市人大常委会审议意见和市政协建议案77件。制定规范性文件365件、废止198件，受理行政复议案件157件。推进政务公开，建成“12345”市长热线、政风行风热线、网络问政等互动平台，五年发布政务信息90多万条，办理群众热线电话40.4万个。掀起效能风暴，加强绩效管理，从严治理庸懒散慢乱。认真落实政府系统党风廉政建设责任制，完善反腐惩防体系，大力纠正不正之风。五年完成审计项目2380个，查办违纪违法案件2284件，处分2437人，移送司法机关134人。

　　2012年是本届政府任期的最后一年。面对宏观经济下行压力，我们紧紧咬住冲刺全省第一方阵的主目标，大力实施“661”行动，深入开展大滁城建设提升年、示范区建设突破年、城乡统筹推进年活动，认真组织千名干部帮扶千家企业、帮抓千项工程、帮招千个项目，努力做到稳中求快、进中求好。一是贯彻执行中央和省委、省政府政策，出台34项叠加扶持措施，落实“营改增”试点及结构性减税政策，促进了实体经济平稳较快发展。二是注重发挥投资对增长的拉动作用，新建续建项目2000个。社会融资总量超300亿元，其中域内外新增贷款170亿元，市城投公司15亿元债券发行到位，市开发区同创公司8亿元债券获批发行，开展住房公积金贷款试点并放贷4.9亿元，天大集团8.6亿元短期融资券成功注册。三是坚定不移招大引强，推进园区建设。新引进亿元以上项目近500个，招商到位资金1300亿元，省级以上园区新增开发面积20平方公里。四是着力推进苏滁产业园、琅琊山和小岗村三个“两年大变样”。苏滁现代产业园一期“八纵六横”30公里路网和34万平方米安置房全面开工，引进工业项目12个。启动琅琊山概念性规划国际招标，中华姓氏牌匾园等重大项目深度推进，冠景五星级酒店开工建设。小岗村总投资20亿元的32个项目全面实施。五是大力推动农业持续稳定发展。滁河、池河防洪近期治理工程开工建设，完成70万亩高标准基本农田整治任务，粮食产量415万吨，8个现代农业示范区建设深入推进。六是加快发展服务业。家电下乡销售额居全省前列，沃尔玛、定远大润发、天长天发广场三期、中信银行、兴业银行、4家村镇银行开业，文化、旅游等产业加快发展。七是全面启动美好新滁城建设。新建续建项目323个、道路119条275公里，完成房屋征收118万平方米，建设安置房276万平方米，完成投资192亿元。定远、凤阳、明光、天长4个县城新建续建项目281个，完成投资119亿元。37个美好乡村示范点规划建设全面展开。徐明高速滁州段等3条高速公路和312线天长段等5条省道改扩建工程加快建设，公用事业投资和房地产开发指标均居全省前列。八是持续扩大民生投入，加强社会建设与管理。33项民生工程投入51.6亿元，全面完成省下达任务。2012年，全市生产总值增长13.1%，财政收入增长21.8%，固定资产投资增长27.9%，规模以上工业增加值增长17.9%，社会消费品零售总额增长16.9%，外贸进出口总额增长35.4%，城镇居民人均可支配收入增长14%，农民人均纯收入增长15.3%，增速均高于全省平均水平。

　　各位代表！2008年以来，我们先后受到国际金融危机、国内宏观形势变化以及冰雪、洪涝、干旱灾害带来的严峻挑战，全市上下坚持把解放思想作为崛起跨越的强大动力，把体制机制创新作为激发活力的有效途径，把改善民生作为推动发展的根本目标，把扎实苦干作为通向成功的关键举措，迎难而上、破难奋进，迎来了我市综合实力提升最快、城乡面貌变化最大、社会建设成果最丰、人民群众得益最多的好时期。在此，我代表市政府，向全市人民，向市人大代表、政协委员，向各民主党派、工商联、无党派人士，向各人民团体、部省驻滁单位和离退休老同志，向驻滁人民解放军、武警官兵、公安政法干警，向所有关心、支持、参与滁州建设和发展的海内外朋友们，致以衷心的感谢和崇高的敬意!

　　在肯定成绩的同时，我们也清醒地认识到，当前滁州经济社会发展中依然存在着不少困难和问题，要素供给上，资金依然供不应求，土地资源日趋紧张，人才短缺，劳动力既有就业不充分、也有企业用工不足的问题。经济发展上，总量不大，产业层次不高，大项目、大企业不多，高新技术和战略性新兴产业的牵动力不足；县域经济大多处于全省中游位置，不强���不均衡现象相较突出；民营经济发展环境有待进一步优化、速度有待进一步提高。城乡建设上，城镇化率低于全省平均水平，中心城市的首位度不够高，城镇的集聚功能不够强，人居和生态环境还不够好，城乡管理尚需切实加强。民生事业上，城乡居民收入不高，特别是城镇居民人均可支配收入仍低于全国、全省平均水平，社会事业尤其是教育、文化、卫生、体育等还存在不少欠账。政府自身建设上，作风与效能建设方面还存在薄弱环节，形式主义、官僚主义以及庸懒散慢奢现象还不同程度存在，反腐倡廉任重道远。这些困难和问题，是客观的，是前进中的，是需要我们高度关切和认真对待的，我们将以负责任的态度，着力加以解决！

　　二、未来五年的总体工作思路

　　当前，滁州加速崛起的通道已经打开，乘势腾飞的局面已经形成，建设美好新滁州的蓝图已经绘就。尽管宏观经济形势比较复杂、区域经济竞争更加激烈、要素保障的压力仍然很大，但是，有党的十八大精神指引，有省委、省政府和市委的坚强领导，有多年蓄积的坚实基础，有全市上下不甘落后、团结奋进的坚定信心和决心，滁州一定能够奋进前行，实现更好、更快、更大发展。未来五年，我们将全面贯彻落实党的十八大精神，以邓小平理论、三个代表重要思想、科学发展观为统领，紧紧围绕主题主线，以冲刺全省第一方阵、打造美好安徽第一印象为主目标，以十大工程、百亿项目、千亿产业为主抓手，坚持“五位一体”，突出“四化同步”，推进“五大战略”，实施“三步走”，开拓创新、苦干实干，确保经济持续健康快速发展，争取主要经济指标全面翻番，基本建成小康滁州。

　　那时候的滁州，经济将走向繁荣。创新能力和信息化水平明显提升，新型工业化将成为经济增长的主导力量，现代服务业和现代农业取得长足发展，全市生产总值达到2200亿元，财政收入达到330亿元，社会消费品零售总额达到700亿元，总体实力迈入全省第一方阵。

　　那时候的滁州，城乡将展现新貌。美好新滁城和美好新县城初步建成，美好乡村达标过半，立体交通网基本形成，环保意识、能力和水平明显提升，城乡环境优美、生态良好、宜居宜业。

　　那时候的滁州，生活将实现富裕。经济的蓬勃发展将为就业创业提供更多的机会，全民创业成为滁州的内生动力和社会潮流，城镇居民人均可支配收入达4.1万元，农民人均纯收入超1.7万元。

　　那时候的滁州，社会将更加和谐。政府公共服务能力和水平显著提高，社会管理不断创新，社会保障明显加强，社会矛盾有效化解，教育、卫生、文化、体育等社会事业长足进步，城乡居民幸福感进一步提升。

　　（一）紧紧把握跨越发展这一第一使命，全力做大做强市域经济。发展仍然是解决一切问题的前提和根本。未来五年，要把扩大投入作为主要举措。工业要在冲刺全省第一方阵中当主力、打头阵，围绕打造千亿产业、百亿企业，新上、技改项目并重，组建工业投资平台，五年力争完成投资3000亿元，其中技改投资1200亿元以上，到2017年，力求10个以上企业超百亿。服务业要充分发挥区位优势，以汊河南京湾等重大项目为龙头���加快辐射带动，按照物流园区、物流中心、物流配送三个层次推进，打造“中国商谷”，五年力争完成投资1000亿元，建成亿元以上项目400个。农业要推进由大向强的转变，实施“1383万千百十”工程，突出现代农业示范区、农业产业化、森林增长工程和水利滁州建设等重点，五年力争完成投资800亿元。同时，全力推进滁宁城际铁路、马滁扬高速、滁淮高速、大唐电厂、江巷水库等一批重大基础设施项目，五年力争完成各类固定资产投资5000亿元。要把转型升级作为关键路径。加速工业化、城镇化、农业现代化与信息化的深度融合，着力改造家电、机械、轻纺、食品、建材等传统产业，大力发展新能源、新材料、电子信息、生物医药等战略性新兴产业，积极发展现代物流、电子商务、金融服务、文化创意、服务外包、养生养老等现代服务业，着力打造在全国有影响力的农业产业化“龙型”经济，建设泛长三角地区新兴的制造业基地、江北重要的物流枢纽、华东地区独特的休闲度假旅游目的地。要加强创新型城市建设，大力培育创新型企业，积极推动产学研合作，加强科技人才引进和培养，全面提升自主创新能力，保障转型升级。要把改革开放作为根本动力。完成国有集体企业改制扫尾，加快现有企业嫁接重组。鼓励、支持、引导非公有制经济发展，强力推动全民创业。深化行政体制改革，加快事业单位分类改革，推动财税、投融资、收入分配、住房保障、文化、教育、医药卫生等领域改革。切实加强对内对外开放，积极融入长三角和上海、南京、合肥三大都市圈，进一步提升利用外资水平，大力发展对外经贸合作，提高经济外向度。要把县域经济作为重要支撑。县域经济不强是我市的短板。要因地制宜走强县之路，切实推动县域经济大发展。积极支持天长率先发展，冲刺全国百强县。积极支持来安与南京江北地区融合发展、全椒打造合宁两大都市圈节点城市、定远融入合肥都市圈、明光参与苏滁经济带建设。积极支持凤阳发挥合芜蚌自主创新区和加快皖北发展政策叠加优势，在沿淮地区加快崛起。

　　（二）紧紧把握示范区建设这一重大机遇，全力推进园区跨越发展。最大限度发挥好皖江示范区的政策效应和品牌效应，借力发展。以苏滁现代产业园为核心，以邻近江苏的19个园区为重点，全力打造苏滁经济带，使之成为滁州乃至全省重要的增长带。强化园区的规划和基础设施建设，突出抓好园区的资源特别是土地资源的整合和集约节约利用，组建投融资平台。以效能提升为中心，创新优化管理机制，推动一批园区争创国家级和省级开发区、高新区，早日成为千亿级、五百亿级园区。争取到2017年，全市园区建成区面积超过350平方公里，税收收入超过150亿元。围绕主导产业招大引强。力争招商引资到位资金年均增长20%以上。转变招商理念和方式，注重全面承接、创新承接、提升承接、有效承接，挑商选资，严把项目投资强度、投入产出和能耗排放关，努力引进研发机构、金融组织、总部经济和高端人才。

　　（三）紧紧把握城乡统筹这一基本路径，全力推进美好新滁州建设。以打造又好又美又时尚的安徽第一印象为目标，精致建设美好新滁城。围绕建设合宁之间新型区域中心城市的战略定位和千年亭城的特色定位，按照一年主攻重点、两年初具规模、三年全面提升、五年基本建成的总体要求，高水平规划，高品质建设。突出抓好一山（琅琊山环山景观带）、一城（古城）、一河（清流河）、一湖（明湖）、一库（土桥水库）、一镇（乌衣古镇）、一路（滁州大道）工程，打造城市亮点和名片。力推苏滁产业园成标杆、琅琊山旅游区大变样、城南新区旺人气、古城区显魅力，基本建成高铁站区和科教园区。继续实施“131”组团发展战略，加速内外两个同城化，将琅琊新区、南谯新城纳入主城区，以全椒、来安两个县城和汊河新城为副中心，扇形展开，协同发展，与南京无缝对接。争取到2017年，“大滁城”建成区面积达到180平方公里，人口达到150万。加强城市管理，全面实现“六城联创”目标。加快建设美好新城镇。按照现代化中等城市标准，改造老城区，提升新城区，扩张产业园区，新老并重、产城一体、建管结合推进各县城建设。按照“镇当城建”的思路，打造一批5-10万人口的镇级小城市和精品小城镇。积极推动农村转移人口市民化，通过努力，使全市城镇化率提高到50%以上。全面推进美好乡村建设。围绕生态宜居村庄美、兴业富民生活美、文明和谐乡风美的目标，从今年起，每年重点培育建设150个左右中心村、治理改造1000个左右自然村。争取到2017年，60%以上中心村达到美好乡村要求。

　　（四）紧紧把握民生改善这一最终目的，全力建设人民安居乐业的幸福城市。完善学前教育，均衡发展义务教育，继续抓好职教大市建设，加快薄弱学校改造步伐，促进教育公平。基本完成城市职业学院建设，积极引进或组建新的大专院校。健全公共医疗卫生服务体系，提升医院、卫生院和社区卫生服务中心建设水平，推进基本公共卫生服务均等化，全面完成公立医院改革。切实加强食品药品安全监管。坚持文化事业与文化产业相互促进，大力实施文化惠民和全民健身工程，鼓励引导文化消费，提高群众文化科学素质。深入实施城乡居民收入倍增计划，坚持就业优先策略，深度开发城乡人力资源，争创国家级创业城市，推动更高质量就业。推动养老、失业、医疗、住房、最低生活等社会保障由广覆盖向全覆盖转变。增加民生投入，创新社会管理，统筹解决人口问题，扎实做好安全生产、社会管理综合治理、信访等工作，努力维护社会稳定，促进社会和谐。

　　（五）紧紧把握生态保护这一基本要求，全力营造高水平的绿色家园。以创建国家级生态示范区、森林城市和低碳城市为抓手，加强生态文明建设。全面完成251万亩森林增长工程。扎实抓好节能减排和污染治理，加强饮用水源、森林资源和湿地、耕地保护。大力发展节能环保产业，积极发展循环经济，促进生产、流通、消费过程的减量化、再利用和资源化。倡导低碳生活，发展公交事业，推行清洁生产，构建资源节约、环境友好型社会，实现人与自然、经济与环境的和谐共生。

　　为了做好以上工作，我们要进一步加强政府自身建设，依法行政要有新进展，转变职能要有新作为，机制创新要有新突破，工作效能要有新提升，为滁州跨越发展提供坚强保障。

　　三、2013年工作安排

　　2013年是新一届政府的开局之年。市政府将抢抓当前宏观经济趋好、宏观政策利好的机遇，紧紧把握稳中求进的总基调，大力实施“661”工程和十项重点工程，奋力攻坚突破，全力推进经济社会平稳较快发展，确保生产总值增长13%，财政收入增长15%，固定资产投资增长25%，社会消费品零售总额增长17%，进出口总额增长16%，城镇居民人均可支配收入增长15%以上，农民人均纯收入增长15%以上，人口自然增长率低于7‰，完成节能减排任务。力争超额完成，实现良好开局。

　　（一）全面推进皖江示范区建设。创新园区管理机制，强化用钱、用地、用工等保障，积极推动城区企业“退二进三”、“退城入园”，力争完成园区基础设施投资100亿元以上，新增开发面积30平方公里。高标准编制苏滁经济带发展规划，协同作战，重点突破。苏滁现代产业园新建续建50个重点项目，其中工业项目10个以上，完成投资30亿元，实现两年大变样。市开发区要聚精会神抓项目，进一步发挥领头羊作用，其规工增加值、财政收入、固定资产投资、招商引资到位资金要争取分别增长40%、40%、50%、50%。天长开发区积极创建国家级开发区。坚持高位推进，驻点招商、网上招商、以商招商、产业链招商多措并举，力争全年新引进亿元以上项目500个以上，市开发区、苏滁现代产业园、市集中示范园区、高铁站区、科教园区、琅琊山景区和各县市区都要力争引进1个以上总投资超过50亿元的项目，全市招商引资到位资金增长20%以上。强化项目服务和保障，签约项目抓开工，开工项目抓投产，投产项目抓达产。

　　（二）大力推动工业经济快速增长。实施国家级家电信息产业基地、专用汽车产业城和省级盐化工、硅基材料、新能源、食品产业基地等规划，狠抓工业投入和项目建设，每个县市区都要精选、动员、组织不少于2家规工企业靠大引强，力争全市工业总产值超过2000亿元。家电信息产业，开工建设德泰挠性覆铜板、全息光伏电池、富达电器、康佳模组线技改等项目，确保博西华新基地、盛诺电子、恒天科技、必祥必拓光电、爱沃富光电、华彩电器、家家爱电器、奥特佳压缩机等项目投产。装备制造产业，开工建设新联塑注塑机、胜华波汽配、先特能源装备、欧迪恩传动、印度汽车零部件等项目，加快建设长丰汽车、永强汽车、福友汽配、凤阳城市矿产等项目。硅玻璃产业，开工建设东方光源光纤光缆、德力施贝乐奶瓶等项目，确保台玻、力诺、淮河玻璃等项目竣工投产。盐化工产业，争取华塑盐化二期、中联能源四期等项目上马建设，确保泉盛化工一期、华纳化工、金禾技改、天大BOPP薄膜等项目建成投产。农副产品精深加工产业，开工建设东鹏饮料、博凯草本咖啡等项目，确保雀巢银鹭、蜡笔小新、雅客食品、喜多多食品、宝利嘉纺织等项目投产。新能源新材料产业，开工建设远东电缆、中澳新能源、德威新材料、远大可建和定远、全椒等风电项目，继续推进天康钛酸锂电池、博特复合材料等项目建设，确保凤阳风电、南谯和来安风光互补发电建成投产。同时，抓好贝发文具城、天虹制药、新兴化纤等项目建设。继续抓好100项年内确保竣工的亿元以上项目，实施300项重点技改工程。全年力争完成工业投资510亿元，其中技改投资220亿元。常态化实施“三千大会战”，切实帮助企业解决落地、建设以及生产、经营中的困难和问题。重视抓好新投产企业稳产达产，争取华塑盐化一期、长电科技、盼盼食品、中联能源三期、确成硅化工等65个新投产项目早日达产。继续实施“三上”工程，争取新增规模以上企业100家。大力推动科技创新。支持中能柔性显示、家电机器人等实现产业化，力争新增创新型和高新技术企业50家、省级以上技术中心6个、博士后流动工作站2个、院士工作站2个，建成国家电子元器件质检中心、来安精细化工产品检验中心和全椒科技孵化器，开工建设来安科技孵化器和天长电线电缆、定远盐化工产品检验中心。

　　（三）深入推进美好新滁州建设。美好新滁城建设，完成新一轮城市规划修编，“131”组团实施建设项目400个，建设道路123条、500公里，新建保障房300万平方米，完成投资300亿元。老城区加快建设南湖四期，开工建设北湖一期、内城河闸和清流河景观带二期等项目。城南新区开工建设龙蟠河文化休闲街区。科教园区重点推进城市职业学院新校区、新宇软件产业园、瑞飞科技园、柔性光电工程研究中心等项目建设。高铁站区重点推进国际五金机电城、月星家居广场、高星级酒店、安置房和路网建设。琅琊山旅游区重点建设醉翁亭核心景区、丰乐亭景区和冠景休闲度假区、中华姓氏牌匾园、东方醉园等项目。琅琊新区进一步充实完善，加快路网和配套设施建设，建成政务新区，谋划启动跨清流河开发。南谯新城重点抓好第五期路网建设和安置房、碧桂园等项目。全椒加快推进城南新区和高铁站南区建设，积极打造儒林、太平两大文化街区。来安连片推进新城区建设，加快汊河新城城乡一体、产城融合的规划建设步伐。启动实施千年亭城计划。积极开展城市管理十大提升行动，推动“六城联创”，提升城市管理水平。美好县城建设，天长市按照《胭山风物图》再现古城景观，努力打造皖东“小扬州”。明光市重点推进东部新区、滨河风光带建设和老城路网改造。凤阳县进一步充实完善新城区，抓好明中都遗址公园和钟楼文化中心建设。定远县要大视野、大思路、大手笔谋划定城发展的大格局，积极开发泉坞山和桑涧水库周边地块，有效衔接城东新区和高铁站区，实现引山引水入城。4个县城新建续建项目269个、力争完成投资198亿元。美好乡村建设，抓好30个示范村、7个示范镇建设，整治1000个自然村，精心打造主城区到全椒、汊河、乌衣3条示范长廊，基本完成小岗村“两年大变样”建设任务。按照规划控制、成片开发、镇当城建的要求，高标准推进秦栏、铜城、炉桥、板桥、女山湖、六镇等重点镇建设，发挥示范引领作用。城乡基础设施建设，加快徐明高速、滁马高速滁州段、蚌淮至宁洛高速连接线和309省道明光段、311省道定远段等工程建设，开工建设宿扬高速天长段、104国道滁汊段改扩建和全椒襄河航道升级等工程，积极推进汊河船闸改建、窑河复航、滁来全第二通道等项目，完成全社会交通投资120亿元。加速电网建设与改造步伐，完成电力投资6.1亿元；积极推进智慧城市建设，完成通信投资5.7亿元。

　　（四）不断加快发展服务业。提升商贸流通。继续抓好汊河南京湾、明光中都商厦、定远北极星购物中心等建设，开工建设创达义乌商贸城、天长月星家居广场、滁州农产品批发市场等项目。加强对进出口企业的扶持，推进海关特殊监管区申报工作，实现对外贸易持续增长。今年新增限额以上商贸企业要超过100户。拓展金融服务。加强银政企合作，扩大社会融资总量，发展各类新型金融组织，确保新增贷款增速超全省平均水平，确保市同创公司8亿元债券近日发行成功，尽早发行一批短期融资券、中期票据、中小企业集合债。积极引进新的风投基金和股份制商业银行，成立凤阳农商行，完善担保体系，支持小额贷款公司扩大规模、规范经营，加速扬子木业和博润电脑、天康股份等上市进程，支持上市公司再融资，培育上市后备资源。继续发展保险业。优化房地产开发结构。积极建设保障房、中小户型商品房，发展商业地产、养老地产、文化地产、旅游地产、工业地产等，确保房地产完成投资240亿元。发展现代物流。加快建设汊河商贸、天长益民、定远盐化等物流园和凤阳港二期等重点物流项目，规划建设全椒—滁州物流集聚区、明光赵府港口物流园，着力引进国内外知名物流企业。实施旅游业突破工程。开展琅琊山景区两年大变样、小岗村旅游品牌提升、特色景区开发、乡村旅游产品打造四大行动，加快建设天长金牛湖、明光栖凤湖和跃龙湖、全椒神山、来安舞彩、定远大金山、凤阳韭山地质公园、南谯古民居和琅琊1912文化街区等重点项目以及港汇喜来登、凤阳凤仪、汊河碧桂园等高星级酒店，结合美好乡村建设，打造一批高水平的农家乐。力促文化和旅游融合，整合资源创办滁州民俗文化旅游节。

　　（五）努力提高现代农业发展水平。大力推进农业产业化经营，加快嘉吉、德青源、全椒辉隆、小岗宝迪等产业化项目建设，推动一批企业争创国家级和省级龙头企业。推动土地依法有序流转，新增流转面积30万亩，促进农业生产规模化、集约化。抓好农业物联网试点，基本建成8个现代农业示范区。发展农业机械化。加强农技推广体系建设，开展粮食高产攻关竞赛，加强防灾减灾和动物疫病防控，扩大农业保险，力争农业再获丰收。继续实施滁河、池河防洪治理和高邮湖大堤加固工程，开工建设总投资30亿元的方邱湖和花园湖淮河行蓄洪区调整等工程，完成8座中型、127座小型水库除险加固。完成95万亩高标准农田建设及1.5万亩土地整治任务。

　　（六）持续推动社会事业进步。全面完成学前教育三年行动计划和101所义务教育学校标准化建设，规范校车运营管理。加快省级职业教育改革试验区和职教大市建设，整合滁城职教资源，创建滁州机电职业学院。巩固完善基层医改，完成县级公立医院改革任务，稳步推进基本公共卫生服务均等化，加强重大疾病防控。启动市图书馆、新广电中心建设项目，谋划市地方志展示馆，争取实现市科技馆、文化馆、博物馆开馆，加快建设县级图书馆、文化馆，建成一批全民健身工程和村文化活动中心。精心办好第五届中国农民歌会和第十三届全国村长论坛。争创全省双拥模范城七连冠。深化计划生育“村为主”机制，稳定低生育水平，推动计生工作再上新台阶。认真实施好城乡居民收入倍增计划。全面推行企业工资集体协商制度。加强就业培训、招工服务和创业型城市建设，统筹化解“就业难”和“用工荒”，力争新增城镇就业5.8万人、个体工商户1万户、民营企业2000家。做好各类社会保险扩面提标工作。发展社会救助和社会福利事业，建设一批乡镇村养老服务中心、社区日间照料中心。开工建设保障房2.66万套，改造农村危房1.5万户。继续实施33项民生工程，确保配套资金及时足额到位。同时，抓好外事、侨务、对台、统计、编制、档案、保密、人防、气象、地震、科普等工作，支持工会、共青团、妇联、工商联、红十字会等工作，发展妇女、儿童、老年人、残疾人等各项事业。

　　（七）进一步加强社会管理。加快乡镇群众工作站和新型农村社区建设，抓好社区管理体制改革试点，打造一批示范社区。开展企业安全生产达标创建，强化源头治理，严厉打击各类违法生产经营活动，坚决遏制重特大安全生产事故发生。深入推进食品安全“五放心”体系和市场物价监测体系建设。开展社会矛盾预警监测和调处工作，畅通信访渠道，解决群众合理合法诉求，努力化解不稳定因素。认真落实民族宗教政策。改进城乡社区警务工作，推进立体化社会管理防控体系建设，加快建设社会管理视频监控系统和智能交通管控设施，加强网络监管和舆论应对，强化对重点人群的服务和管理，严厉打击违法犯罪，建成市应急指挥中心，妥善处置公共突发事件，保持社会和谐稳定。

　　（八）扎实抓好生态建设。首战必胜，完成54.8万亩造林任务。严把项目环评、能评准入关，淘汰落后产能。狠抓水污染防治，建成来安、明光污水处理厂二期和南谯新城、汊河新区污水处理厂等工程，全面完成西涧湖饮用水源地环境综合治理，对来河、襄河、清流河、滁河等河流污染进行整治。狠抓机动车尾气减排，加强局部地区烟尘、粉尘危害治理。完成4个农村环境治理项目，将农村垃圾“村收集、乡转运、县处理”范围扩大到70个乡镇。加大矿产资源整合力度。抓好市垃圾焚烧发电和明光、凤阳生物质发电等项目建设，继续推广使用农村沼气，分类整治利用工矿废弃地，推进矿山修复工程。

　　（九）切实加强政府自身建设。坚持依法行政。深入开展“六五”普法和依法行政单位创建活动，推进依法行政工作。自觉接受人大工作和法律监督，自觉接受政协民主监督，虚心听取各民主党派、工商联、无党派人士和各人民团体的意见，认真办理人大议案、代表建议和政协提案，主动接受人民群众和媒体舆论监督。深化政务公开。完善重大决策程序，开展决策社会风险评估，进一步办好“12345”市长热线，实施网络问政，扩大社会听证，拓宽社情民意表达渠道。规范政务公开程序，丰富公开形式，加大重点信息公开力度，大力推进基层政务公开。切实改进作风。严格执行中央八项规定和省三十条要求，坚持实干兴滁，采取有力措施，下决心解决“文山会海活动多”等问题，坚决克服形式主义、官僚主义。不断提升效能。抓好政府系统干部职工的学习和教育，不断增强服务意识，提高服务能力。深化行政审批制度改革，推进基层行政服务中心建设，加大企业帮扶帮办力度。强化督查问责，狠抓绩效考评，促进效率提高。加强廉政建设。严格落实政府系统党风廉政建设责任制，强化对公共资源交易和招投标等重点领域监管，加强政府投资项目资金管理和跟踪审计，严肃查处违纪违法案件，始终保持惩治腐败的高压态势。开展节约型机关建设，严控一般性支出，把有限财力用在发展经济和改善民生上。

　　各位代表，新的目标催人奋进，新的征程已经开启，使命光荣，任务艰巨。让我们紧密团结在以习近平同志为总书记的党中央周围，在省委、省政府和市委的坚强领导下，振奋精神，锐意拼搏，扎实工作，为加快建设美好滁州、全面建成小康社会而努力奋斗！
